# Release of chromatin extracellular traps by phagocytes of Atlantic salmon, *Salmo salar* (Linnaeus, 1758)

**DOI:** 10.1016/j.fsi.2021.08.023

**Published:** 2021-12

**Authors:** Neila Álvarez de Haro, Andre P. Van, Calum T. Robb, Adriano G. Rossi, Andrew P. Desbois

**Affiliations:** a Institute of Aquaculture, Faculty of Natural Sciences, University of Stirling, Stirling, FK9 4LA, United Kingdom; b University of Edinburgh, Centre for Inflammation Research, Queen's Medical Research Institute, Edinburgh, EH16 4TJ, United Kingdom

**Keywords:** ETosis, Macrophage, NETosis, Neutrophil extracellular traps, Polymorphonucleocyte

## Abstract

Neutrophils release chromatin extracellular traps (ETs) as part of the fish innate immune response to counter the threats posed by microbial pathogens. However, relatively little attention has been paid to this phenomenon in many commercially farmed species, despite the importance of understanding host-pathogen interactions and the potential to influence ET release to reduce disease outbreaks. The aim of this present study was to investigate the release of ETs by Atlantic salmon (*Salmo salar* L.) immune cells. Extracellular structures resembling ETs of different morphology were observed by fluorescence microscopy in neutrophil suspensions *in vitro*, as these structures stained positively with Sytox Green and were digestible with DNase I. Immunofluorescence studies confirmed the ET structures to be decorated with histones H1 and H2A and neutrophil elastase, which are characteristic for ETs in mammals and other organisms. Although the ETs were released spontaneously, release in neutrophil suspensions was stimulated most significantly with 5 μg/ml calcium ionophore (CaI) for 1 h, whilst the fish pathogenic bacterium *Aeromonas salmonicida* (isolates 30411 and Hooke) also exerted a stimulatory effect. Microscopic observations revealed bacteria in association with ETs, and fewer bacterial colonies of *A. salmonicida* Hooke were recovered at 3 h after co-incubation with neutrophils that had been induced to release ETs. Interestingly, spontaneous release of ETs was inversely associated with fish mass (p < 0.05), a surrogate for age. Moreover, suspensions enriched for macrophages and stimulated with 5 μg/ml CaI released ET-like structures that occasionally led to the formation of large clumps of cells. A deeper understanding for the roles and functions of ETs within innate immunity of fish hosts, and their interaction with microbial pathogens, may open new avenues towards protecting cultured stocks against infectious diseases.

## Highlights

• Chromatin extracellular traps (ETs) were released by salmon neutrophils.
• Calcium ionophore was a powerful inducer of ET release from neutrophils.
• ETs were decorated with histones H1 and H2A and neutrophil elastase.
• Bacteria induced ET release and association with ETs was observed.
• ET structures were observed in monocyte/macrophage suspensions.

## Graphical abstract

**Figure:**
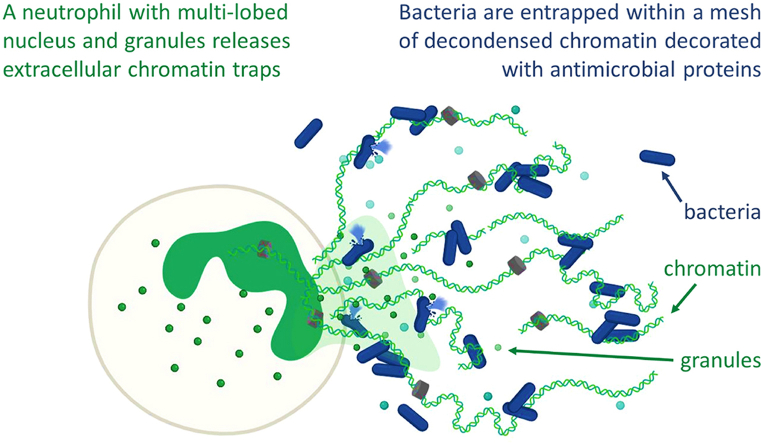

## 1 Introduction

Various cell types recognise and respond to microbial invaders as part of the fish innate immune response. The polymorphonuclear cells (PMNs; also known as granulocytes), such as neutrophils, basophils and eosinophils, play a particularly important role and they act through a series of mechanisms to counter microbial threats [1,2]. PMNs, particularly neutrophils, perform phagocytosis, where microbes are internalised and inactivated in phagosomes by reactive oxygen species (ROS) and other antimicrobial compounds [[3], [4], [5]]. Like other granulocytes, neutrophils release antimicrobial compounds into the extracellular space by the process of degranulation where they can then act against their targets [1,6]. Finally, neutrophils can release extracellular traps (ETs), which are structures composed of decondensed nuclear chromatin embedded with antimicrobial proteins, including neutrophil elastase, myeloperoxidase (MPO) and histone fragments [[7], [8], [9], [10], [11]]. ETs exert their antimicrobial action by trapping microbes to prevent or delay their dissemination around the host sufficiently to allow for other immune cells to be recruited to assist in preventing an infection [12,13]. Moreover, ETs may themselves be directly antimicrobial because the chromatin structure provides a means to bring the microbes into close proximity with the decorating proteins such as neutrophil elastase, MPO and histone fragments that can exert these activities [[14], [15], [16]].

Much of the knowledge on ETs derives from studies of mammals and relatively few studies have examined the phenomenon in fish hosts [17]. Still, ETs have been observed to be released by PMNs of fathead minnow (*Pimephales promelas*) [18], zebrafish (*Danio rerio*) [19], common carp (*Cyprinus carpio*) [20,21], turbot (*Scophthalmus maximus*) [22], sole (*Cynoglossus semilaevis*) [16], rainbow trout (*Oncorhynchus mykiss*) [10] and Atlantic salmon (*Salmo salar*) [23]. In addition, Pijanowski et al. [24] detected ETs in monocyte/macrophage preparations from common carp, thus demonstrating other immune cells to be capable of releasing the traps. Even with these studies, knowledge of teleost ETs remains nascent and differences in responses observed between species remain to be understood, including the pathways controlling initiation and release, the interaction with and effects on microbes, and the range of cell types capable of this response. Moreover, there is little understanding for variability observed between individual fish in their propensity to release ETs [10].

Though there is increasing recognition for the importance of ETs in humans and other mammals in immunity and the negative impacts associated with dysfunctional control of their release, few of the economically most important fish species have yet to be investigated for the existence, control and role of ET release. Despite a recent observation of ET release in Atlantic salmon [23], a species farmed intensively in the waters of northern Europe and in Chile, very little is known in particular for the constituents decorating the ET structures and the compounds that modulate ET release. Global production of *S. salar* reached 2.4 million tonnes in 2018 [25] and the success of this industry is underpinned by the ability to prevent and control diseases, particularly those caused by bacteria. Vaccination programmes have contributed significantly to infection control but outbreaks requiring antibiotic therapy can still occur where a vaccine does not confer protection [26,27]. However, antibiotic use can increase the risk of selecting for resistant strains that has associated detrimental consequences [[28], [29], [30]], and a better understanding of the salmon innate immune system may uncover alternative solutions to disease prevention and control.

Therefore, the aim of this present study was to investigate the release of ETs by Atlantic salmon neutrophils and other immune cells, including the detection of characteristic components of the structures, the actions of various modulators on release, and the effectiveness of the traps against bacteria.

## 2 Materials and methods

### 2.1 Fish and isolation of immune cells

Apparently healthy pre-smolt salmon (determined by gross examination) were used in all experiments. Fish were maintained at the Niall Bromage Freshwater Research Unit (University of Stirling) in 25 m^3^ tanks at 30–50 kg/m^3^, with the system operating at a flow rate of 50–70 l/min, oxygen concentration >7 mg/l, and typical mean monthly water temperature of 8.3 ± 1.3 °C (±standard error, SEM). Constant light was provided post-hatch from January until switching to a natural photoperiod in July, and fish were fed a commercial pelleted feed (Skretting, Stavanger, Norway) *ad libitum*. Typically, fish were ca. 30–80 g but larger fish (100–300 g) were also sampled occasionally. PMNs and mononuclear leukocytes (i.e., monocytes/macrophages) were isolated from head kidney tissue according to the triple-layer Percoll gradient procedure of Van et al. [10]. A band enriched for PMNs formed at the interface of the 1.060 and 1.072 g/ml Percoll layers, whilst the mononuclear leukocytes collected at the interface of the 1.072 and 1.084 g/ml Percoll layers. Peripheral blood leukocytes (PBLs) were isolated from blood collected aseptically from the caudal vein using a 2.5-ml syringe and 25G × 5/8 needle (Terumo, Surrey, UK). Blood was diluted 5-fold in RPMI-1640 medium supplemented with 40 U/ml heparin and loaded onto a 54% Percoll layer. PBLs were collected from the interphase region. All cell types were washed with refrigerated RPMI-1640 for 10 min prior to use in experiments. Aliquots of washed cells were used for total cell counts, determinations of viability, and Rapi-Diff II staining (Vetlab Supplies, Ltd., Pulborough, UK) to visualise cell morphologies. PBL suspensions were also used for cell sorting and purity assessments by immunostaining (see Section 2.9). Cell suspensions were adjusted to 4 × 10^5^ cells/ml and seeded into cell culture plates (Cell+; Sarstedt, Nümbrecht, Germany) containing RPMI-1640 medium supplemented with 1% (v/v) fetal bovine serum (FBS; Thermo Fisher Scientific, Loughborough, UK) and 0.5% (v/v) penicillin-streptomycin (10,000 U/ml penicillin and 10 mg/ml streptomycin; Sigma-Aldrich Ltd, Gillingham, UK) and maintained at 15 °C for the further experimental procedures.

### 2.2 ET release assay

When quantifying ET release, 50 μl of cell suspension (PMNs, mononuclear leukocytes or PBLs) was seeded at 4 × 10^5^ cells/ml in 96-well cell culture plates and allowed to settle for 30 min at 15 °C before being used for experiments. Blank control wells (lacking cells) contained RPMI-1640 medium and corresponding volumes of diluents only. Plates were incubated at 15 °C (typically for 1 h), before staining for 20 min with Sytox Green (a nucleic acid-specific dye; Invitrogen, Loughborough, UK) that was added to a final well concentration of 5 μM to permit quantification of the fluorescence in each well according to Van et al. [10]. Visualisation of ETs was performed with an Olympus BX-51 epifluorescence microscope equipped with an Axiocam MRC camera (Zeiss, Cambridge, UK) and the Axiovision imaging software (v.4.8; Zeiss).

### 2.3 DNA composition of the ET structures

To confirm the DNA composition of the material released from the cells, PMN suspensions (prepared in 96-well plates as described in Section 2.2) containing cells that had released ET structures spontaneously were incubated with 200 U/ml DNase I (prepared in the buffer containing MgCl_2_ supplied with the enzyme; Thermo Fisher Scientific) for 30 min and stained with Sytox Green as before. Differences in fluorescence were quantified in wells between treatment and controls with a microplate reader and changes in cell morphology were assessed by fluorescence microscopy. Moreover, by combining fluorescence and phase-contrast microscopy, the relative percentages of cells having undergone ETosis was calculated as a percentage of total cells (i.e., viable, dead and ETotic cells).

### 2.4 Effects of chemical and biological modulators of ET release

The effects on ET release of a range of chemical and biological compounds known to modulate ET release in fish and mammals was investigated by exposing PMN and mononuclear leukocyte cell suspensions (prepared in 96-well plates as described in Section 2.2) to calcium ionophore A23187 (CaI; Thermo Fisher Scientific), phorbol 12-myristate 13-acetate (PMA; Sigma Aldrich Ltd, Gillingham, UK), lipopolysaccharide (LPS from *E. coli* O111:B4; Sigma Aldrich), polyinosinic–polycytidylic acid sodium salt (Poly I:C; Sigma Aldrich; PMNs only) and diphenyleneiodonium chloride (DPI; Sigma Aldrich), and ETs quantified by fluorescence according to Section 2.2. The final concentrations added to the wells of the 96-well plates were 5 μg/ml CaI, 10 nM PMA, 10 μg/ml LPS, 40 μg/ml Poly I:C, or 10 μM DPI.

### 2.5 Immunofluorescence antibody test (IFAT)

To characterise the decoration of the chromatin composing the ETs, a panel of antibodies was evaluated against conserved markers of ETs. Specifically, these were mouse to human histone H2A (L88A6) (#3636; Cell Signaling Technology, London, UK), mouse to human histone H1/DNA (MAB3864; Millipore, Watford, UK), and rabbit to human neutrophil elastase (ab21595; Abcam, Cambridge, UK). Secondary conjugated antibody (Alexa Fluor 488) and 4′,6-diamidine-2′-phenylindole dihydrochloride (DAPI) nuclear dye were sourced from Invitrogen. PMNs prepared in 96-well cell culture plates as described in Section 2.2 were induced with 5 μg/ml CaI or 10 nM PMA (1 h, 15 °C) to release ETs and then fixed with 4% paraformaldehyde in PBS for 20 min at room temperature. After fixing, cells were washed with PBS and non-specific binding was blocked with 3% bovine serum albumin (BSA; Merck, Watford, UK) in PBS for 1 h. Then, primary antibodies were added at appropriate dilutions (histone H2A, 1:200; H1/DNA, 1:200; neutrophil elastase, 1:20) and left to incubate overnight at 4 °C. After three washes with PBS, conjugated antibodies (diluted 1:300 in PBS) supplemented with 3% BSA were added for 90 min. Cells were washed thrice with PBS to remove excess antibodies and then incubated with 300 nM DAPI to counterstain the DNA present. Images were acquired immediately with an EVOS FL cell imaging system (Thermo Fisher Scientific) equipped with bright field, 357/44 nm and 470/22 nm excitation LED lights, × 20 and × 40 objective lenses, and processed using the NIS-Elements 3.2 software.

### 2.6 Effect of bacteria on ET release by PMNs

To assess whether bacteria could induce ET release, 100 μl of PMN suspension prepared as Section 2.1 was seeded at 4 × 10^5^ cells/ml in 24-well cell culture plates (Cell+; Sarstedt, Nümbrecht, Germany) and then 100 μl of either of two *Aeromonas salmonicida* subsp. *salmonicida* isolates (*A. salmonicida* Hooke or *A. salmonicida* 30411) was added to the wells to 4 × 10^7^ colony-forming units (CFU)/ml. The bacteria had been cultured from single colonies in 5 ml tryptic soy broth (TSB; Oxoid, Basingstoke, UK) overnight at 22 °C with orbital shaking at 150 rpm. Bacteria were harvested in exponential phase by centrifugation (2600×*g*, 15 min, 4 °C), washed once with PBS and resuspended to the desired CFU/ml. Control PMN cultures were incubated with RPMI-1640 medium and diluent only. After centrifugation (800×*g*; 10 min; 22 °C) to allow the bacteria to come into contact with the cells and materials at the bottom of the wells, cultures were incubated for 2 h at 22 °C. Following this, Sytox Green was added to the cultures to a final well concentration of 5 μM for 20 min, before the fluorescence of each well was quantified with a microplate reader and well contents observed under the fluorescence microscope as above.

### 2.7 Bacteria interaction with ETs

To visualise the interaction between the bacteria and the ETs, PMNs at 4 × 10^5^ cells/ml in the wells of a 24-well cell culture plate were induced to release ETs by exposure to 5 μg/ml CaI for 1 h at 15 °C, before staining with 5 μM Sytox Green as above. Meanwhile, washed bacterial suspensions of *A. salmonicida* Hooke or 30411 were stained with 300 nM DAPI for 30 min at 4 °C, washed with PBS at 2600×*g* for 10 min and resuspended in PBS. Then, the bacterial suspensions were added to the PMN cell suspensions to ca. 4 × 10^7^ CFU/ml. After centrifugation as described in Section 2.6, the cultures were incubated at 22 °C for 2 h and then well contents were observed under the fluorescence microscope with images acquired as above.

### 2.8 Effect of ETs on bacterial viability

To assess the effect of the ETs on bacterial viability, PMN cell suspensions were prepared and induced to release ETs as described in Section 2.7, and then *A. salmonicida* Hooke was added to ca. 4 × 10^7^ CFU/ml. In controls, the PMNs were incubated for 30 min in medium supplemented with 200 U/ml DNase I to digest away any ETs present before bacteria were added. Plates were centrifuged for 10 min at 800×*g* to encourage contact between the bacteria and the ETs, before a sub-sample was taken to determine CFU/ml (time = 0) by serial dilution in PBS and plating on TSA to allow for colony counts. The plate containing bacteria and PMNs was incubated for 3 h at 22 °C at which point the contents of each well were collected and plated to determine CFU/ml. Preliminary studies had determined that DNase I and CaI at the concentrations used had no effect on bacteria viability (data not shown).

### 2.9 Magnetic-activated cell sorting (MACS)

Specific monoclonal antibodies mAb-8 and mAb-42 raised against membrane glycoproteins of trout thrombocytes [31] were provided kindly by Dr Bernd Köllner (Institute of Immunology, Friedrich-Loeffler Institute, Riems, Germany). Atlantic salmon PBLs purified from blood according to Section 2.1 were resuspended in an Eppendorf tube to a density of 4 × 10^5^ cell/ml and incubated with 0.4 μg/ml of mAb-8 and mAb-42 for 30 min on ice. After washing twice with RPMI-1640, cells were resuspended in 160 μl culture medium plus 40 μl of MACS microbeads coupled to a goat anti-mouse IgG antibody (Miltenyi Biotec, GmbH, Germany) for 30 min at 4 °C. After washing twice more, samples were resuspended in RPMI-1640 containing 2 mM ethylenediaminetetraacetic acid and 10% FBS and then loaded onto a mini MACS column (Miltenyi Biotec, Bergisch Gladbach, Germany) to purify antibody-positive cells. Unlabelled leukocytes flowing through the column were discarded. After one wash step with RPMI-1640, the column was detached from the magnetic separator and the bound cells were eluted with 1 ml of culture medium. The thrombocyte-enriched population was quantified with a haemocytometer by light microscopy, and this suspension was used to assess the effects of CaI and PMA on ET release, with assays prepared and performed as described in Sections 2.2, 2.4. To assess purity of the original cell suspension before MACS, an aliquot was incubated with either mAb-8 and mAb-42 and stained with Alexa Fluor 488 goat anti-mouse IgG for 90 min, with 300 nM DAPI used to counterstain the cell nuclei before evaluation by fluorescence microscopy.

### 2.10 Statistical analyses

All statistical analyses were conducted and data plotted with GraphPad Prism v.7.04 software. Spearman's rank and Pearson coefficient were used to assess the significance of the correlations between fish mass and yield of PMNs or the log_10_ of fluorescence values resulting from spontaneous ET release, respectively. Differences between treatment groups in experiments were assessed by Student's t-test (two-tailed). Statistical significance was achieved at p < 0.05, with multiple comparisons accounted for by Holm's correction.

### 2.11 Ethics statement

All procedures were conducted in accordance with the European Directive 63/2010/EU on the protection of laboratory animals used for scientific purposes. The study was approved by the Ethics Committee of the Institute of Aquaculture at the University of Stirling. Euthanasia of the fish was performed in accordance with Schedule 1 of the Animals (Scientific Procedures) Act 1986 (United Kingdom).

## 3 Results

### 3.1 ET structures released by salmon PMNs

**Fig. 1:**
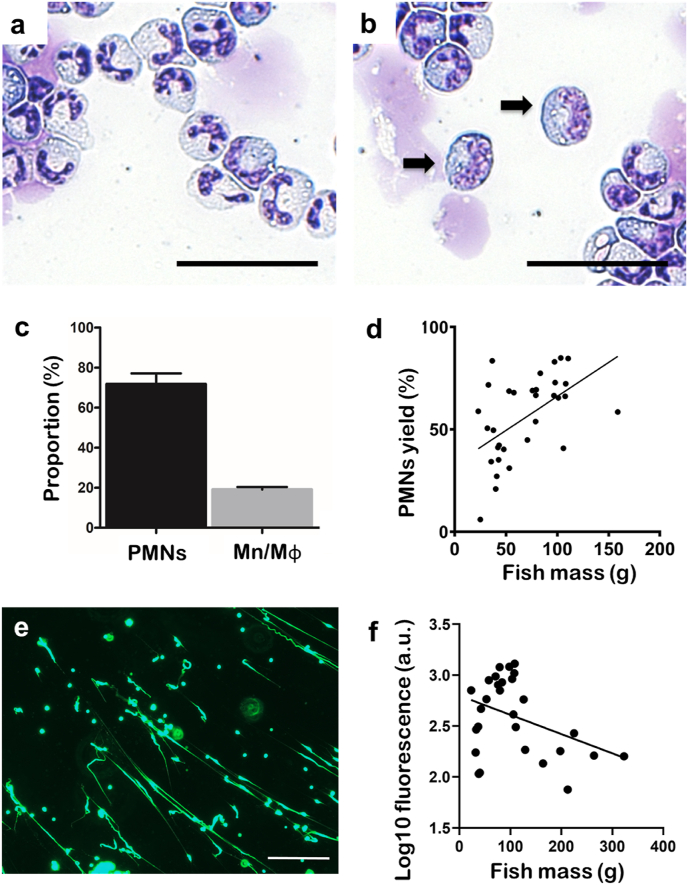
**Neutrophil-like cells isolated from Atlantic salmon head kidney spontaneously released structures resembling extracellular traps (ETs)**. The neutrophil-enriched cell fraction collected at the interface of the 1.060 and 1.072 g/ml Percoll layers was non-adherent in culture and characterised by cytology. **a**. Representative cytological spin slide of the isolated cells stained with Rapi-Diff II. Cells presented a characteristic eosinophilic polymorphonuclear morphology, with the nucleus divided into several lobes, and granulocytic cytoplasm; scale bar, 50 μm. **b**. Magnification of an optical field showing mononuclear cells infiltrating the cytology sample, with arrows indicating cells with mononuclear morphology; scale bar, 50 μm. **c**. Bar chart showing percentages of the different myeloid leukocyte subsets in the isolated cell population (mean ± SEM, n = 6 fish). Polymorphonuclear cells (PMNs; i.e., neutrophils) were the predominant subset (>70%) in the isolated population, followed by monocyte/macrophages (Mn/Mɸ). **d**. Correlation between the mass of each fish and yield of PMNs (mean percentage in each cell preparation) obtained from individual fish (*r*_s_ = 0.4824, p (two-tailed) = 0.0052, n = 32). **e**. Fluorescence microscopy image of neutrophil-enriched cell fraction stained with 5 μM Sytox Green, showing the spontaneous release of ETs *in vitro* after incubation (1 h, 15°C); scale bar, 100 μm. **f**. Association between the mass of each fish and the spontaneous release of ETs as measured by fluorescence after incubation for 1 h at 15°C (log_10_ values used due to heteroscedasticity and non-linear decay shape to curve); r = −0.3814, p (two-tailed) = 0.0452, n = 28. (For interpretation of the references to colour in this figure legend, the reader is referred to the Web version of this article.)

Preparations of cells isolated from the head kidney were highly-enriched in PMNs (Fig. 1); these cells were non-adherent in culture, round in appearance, and contained a polymorphic nucleus surrounded by granulocytic and basophilic cytoplasm (Fig. 1), which are characteristics consistent with mammalian neutrophils. The PMN cell suspensions contained few other phagocytes (Fig. 1). Fish mass correlated directly and significantly with the proportion of PMNs in the cell suspensions (p < 0.05) (Fig. 1).

Web-like and streaky extracellular structures that stained positively with Sytox Green and resembling ETs were observed in PMN suspensions during culture *in vitro*, presumably resulting from spontaneous release (Fig. 1). Interestingly, spontaneous release of ETs (as quantified by fluorescence) was inversely associated with fish mass (p < 0.05) and there appeared to be greater variability in response in the suspensions from fish <120 g (Fig. 1).

**Fig. 2:**
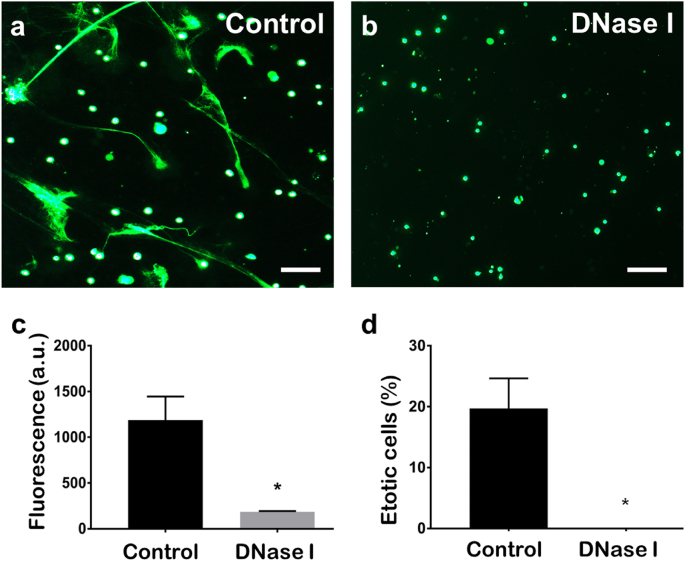
**Nucleic acid structure of the extracellular trap (ET)-like structures confirmed by enzymatic digestion with DNase I. a−b.** Fluorescence microscopy images of neutrophil-enriched cell suspensions from Atlantic salmon cultured *in vitro* and stained with 5 μM Sytox Green. **a**. After settling (30 min, 15 °C), control cells were incubated with RPMI-1640 culture medium for 30 min; scale bar, 100 μm. **b**. The nucleic acid nature of the structures was confirmed by degradation with medium containing 200 U/ml DNase I for 30 min; scale bar, 100 μm. **c**. Bar chart of fluorescence (mean ± SEM) of neutrophil-enriched cell suspensions incubated with culture medium lacking or supplemented with 200 U/ml DNase I for 30 min and stained with 5 μM Sytox Green; ∗ indicates a significant difference from the untreated control (t = 3.86046, p = 0.0048, n = 5). **d**. Bar chart showing the percentage of ETotic cells (mean ± SEM) in the neutrophil-enriched cell suspension following incubation with culture medium lacking or supplemented with 200 U/ml DNase I for 30 min; ∗ indicates a significant difference from the untreated control (t = 3.8871, p = 0.0177, n = 3; percentage data were arcsine transformed before statistical testing). (For interpretation of the references to colour in this figure legend, the reader is referred to the Web version of this article.)

When the PMN suspensions were incubated with DNase I, there was a significant decrease in the fluorescence signal detected in the wells and the web-like structures were no longer observed by microscopy (Fig. 2). Indeed, the percentage of cells that had released observable ETs in the PMN population following treatment with DNase I was reduced significantly compared to untreated controls (Fig. 2).

**Fig. 3:**
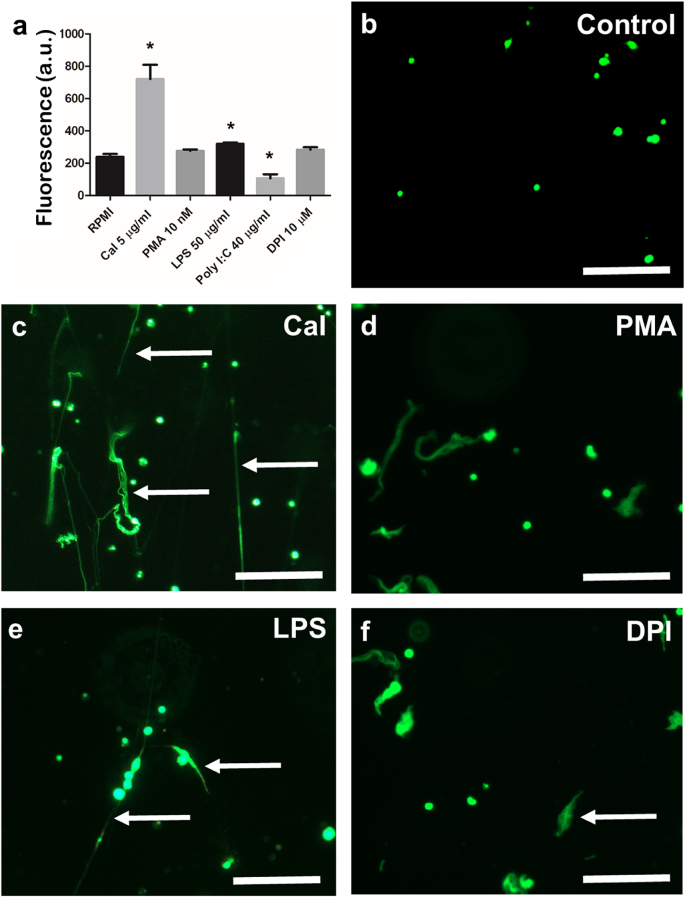
**Effects of previously characterised stimulants on extracellular traps (ETs) released from neutrophil-enriched cell suspensions from Atlantic salmon.** a. Bar chart showing fluorescence (mean ± SEM) of neutrophil-enriched cell suspensions after incubation (1 h, 15 °C) with various proposed inducers and inhibitors of ETosis and stained with 5 μM Sytox Green showing cultures exposed to calcium ionophore (CaI) and LPS had significantly greater fluorescence compared to the untreated control (indicated by ∗; CaI: *t* = −5.3387, p = 0.0007, n = 5; lipopolysaccharide [LPS]: *t* = −3.9831, p = 0.0040, n = 5), which was not the case for phorbol 12-myristate 13-acetate (PMA) and diphenyleneiodonium chloride (DPI) (PMA: *t* = −1.7787, p = 0.1132, n = 5; DPI: *t* = −1.8394, p = 0.1031, n = 5), whilst exposure to polyinosinic–polycytidylic acid sodium salt (Poly I:C) yielded a significant reduction in fluorescence (*t* = 4.2372, p = 0.0028, n = 5). **b−f**. Fluorescence microscopy images of neutrophil-enriched cell suspensions incubated with different compounds (1 h, 15 °C); scale bars, 100 μm. **b.** Untreated controls. **c.** Incubation with 5 μg/ml CaI. **d.** Incubation with 10 nM PMA. **e.** Incubation with 50 μg/ml LPS. **f.** Incubation with 10 μM DPI. Note that the Poly I:C treatment image resembled closely the untreated controls (not shown). (For interpretation of the references to colour in this figure legend, the reader is referred to the Web version of this article.)

Fluorescence was quantified in PMN-enriched cell cultures exposed to various modulators of ET release (Fig. 3). There was a significant increase in the fluorescence of PMNs treated with 5 μg/ml CaI for 1 h compared to the untreated control (Fig. 3). Fluorescence microscopy confirmed the presence of structures resembling ETs in the wells in this treatment group, and these exhibited different morphologies and stained positively with Sytox Green (Fig. 3). Furthermore, there was a small but significant increase in fluorescence in wells containing PMNs exposed to 10 μg/ml LPS for 1 h, while exposure to 40 μg/ml Poly I:C led to a significant decrease in fluorescence compared to untreated controls. Exposure of PMN cell suspensions to 10 nM PMA or 10 μM DPI for 1 h showed no significant difference in fluorescence compared to untreated controls, and this was consistent with microscopy observations that revealed the presence of few ETs (Fig. 3).

### 3.2 Detection of conserved markers of ETs

**Fig. 4:**
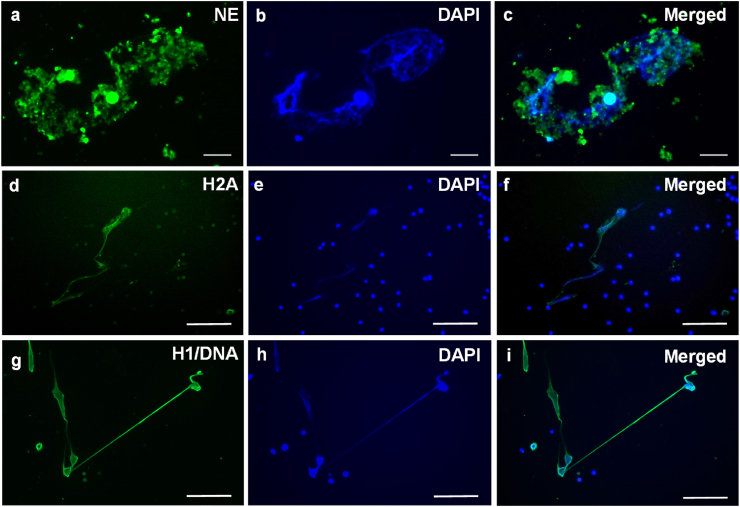
**Confirmation of decoration of the extracellular trap (ET)-like structures with characteristic protein signatures by immunostaining.** Fluorescence microscopy images of neutrophil-enriched cell suspensions from Atlantic salmon cultured *in vitro* with 5 μg/ml CaI (**a**–**f**) or 10 nM PMA (**g**–**h**) for 1 h at 15 °C. **a**–**c.** Immunocytochemical detection of neutrophil elastase in ETotic neutrophils; scale bars, 50 μm. **a.** Localisation of neutrophil elastase by rabbit to human neutrophil elastase and stained with conjugated Alexa Fluor 488 (green). **b.** DNA stained blue with 4′,6-diamidino-2-phenylindole (DAPI). **c**. Merge of a–b. **d**–**f.** Immunocytochemical detection of histone H2A in different ETotic neutrophils; scale bars = 100 μm. **d.** Localisation of histone H2A by mouse to human histone H2A and stained with conjugated Alexa Fluor 488. **e.** DNA stained with DAPI. **f.** Merge of d–e. **g**–**h.** Immunocytochemical detection of histone H1 with DNA in an extended extracellular strand interlinking two cells; scale bars, 100 μm. **g.** Localisation of H1 with DNA by mouse to human histone H1/DNA and stained with conjugated Alexa Fluor 488. **h.** DNA stained with DAPI. **i.** Merge of g–h. (For interpretation of the references to colour in this figure legend, the reader is referred to the Web version of this article.)

Immunofluorescence studies to analyse the composition of the chromatin structures released from salmon PMNs confirmed that these were decorated with proteins characteristic of ETs in other species (Fig. 4). Neutrophil elastase, a major component of the granules in neutrophils, was observed throughout the complex structure of the extruded chromatin (Fig. 4). The nuclear protein, histone H2A, co-localised with the DNA (staining positive with DAPI) in the extracellular environment, confirming a nuclear origin for the chromatin (Fig. 4). We also observed co-localisation of histone H1/DNA in the extracellular strands by using a specific antibody directed against this immunogen (Fig. 4).

### 3.3 Induction of ET release from PMNs by *A. salmonicida*

**Fig. 5:**
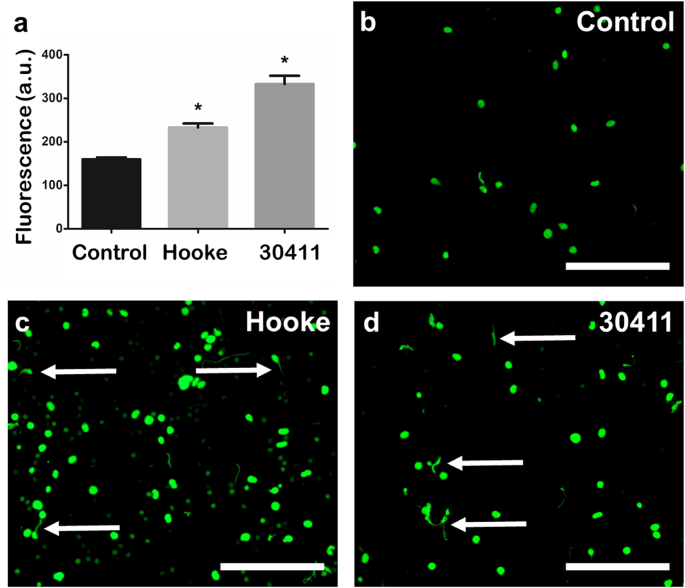
***Aeromonas salmonicida* induced extracellular trap release in neutrophil-enriched cell suspensions from Atlantic salmon.** After settling (30 min, 15 °C), neutrophil-enriched cell suspensions were incubated with *A. salmonicida* 30411 or Hooke at a multiplicity of infection of ca. 100 colony-forming units for 2 h at 22 °C. **a.** Bar chart showing fluorescence (mean ± SEM) of neutrophil-enriched cell suspensions incubated with bacteria and stained with 5 μM Sytox Green showing cultures exposed to *A. salmonicida* Hooke and 30411 had significantly greater fluorescence compared to the untreated control (indicated by ∗; Hooke: *t* = 7.1644, p = 0.0000, n = 6; 30411: *t* = 8.8521, p = 0.0000, n = 6). **b**−**d.** Fluorescence microscopy images of neutrophil-enriched cell suspensions after staining with 5 μM Sytox Green; scale bars, 100 μm. **d.** Untreated control neutrophil-enriched cell suspensions contained few ETs. **c.** Extracellular chromatin was extruded by neutrophils after incubation with *A. salmonicida* Hooke. **d.** Extracellular chromatin was extruded by neutrophils after incubation with *A. salmonicida* 30411. (For interpretation of the references to colour in this figure legend, the reader is referred to the Web version of this article.)

Next, the ability of live bacteria to induce ET release from salmon PMNs was examined. Microscopy observations indicated that PMNs extruded the characteristic web-like structures after interaction with two isolates of *A. salmonicida* and, compared to untreated controls, there was a significant increase in fluorescence in wells containing PMNs exposed to *A. salmonicida* Hooke (233 ± 22.5 a.u.) and *A. salmonicida* 30411 (332.7 ± 46.5 a.u.) (Fig. 5).

### 3.4 Effect of ETs on bacteria viability

**Fig. 6:**
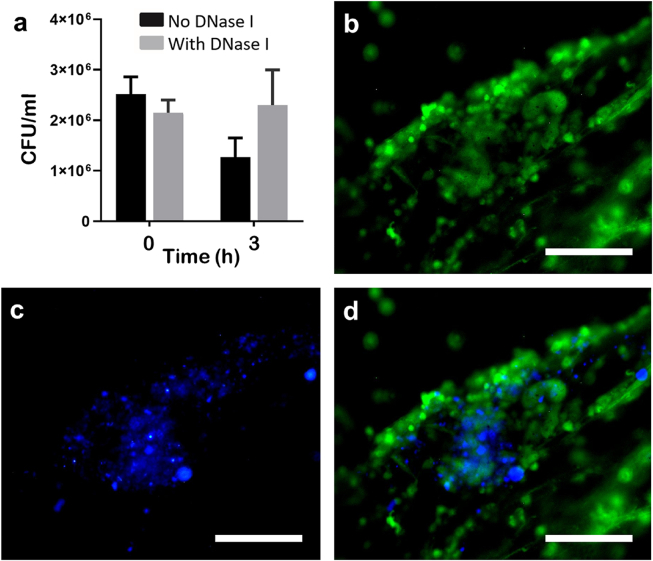
**Interaction between bacteria and extracellular traps (ETs).** Neutrophil-enriched cell suspensions from Atlantic salmon were incubated with 5 μg/ml calcium ionophore (1 h, 15 °C) to induce ET release and then *Aeromonas salmonicida* Hooke was added at a multiplicity of infection of ca. 100 colony-forming units (CFU). **a**. Bar chart showing CFU (mean ± SEM) recovered from the wells containing ETs (No DNase I) or that had been digested away with 200 U/ml DNase I for 30 min (With DNase I) showing there was no significant change in CFU/ml for either treatment at 3 h when accounting for multiple comparisons (No DNase I: *t* = 2.6311, p = 0.0301, n = 5; With DNase I: *t* = −0.2414, p = 0.8153, n = 5). **b**–**d.** Neutrophil-enriched cell suspensions were incubated with *A. salmonicida* Hooke for 2 h and then their interaction was visualised by fluorescence microscopy; scale bars, 100 μm. **b**. DNA was stained with 5 μM Sytox Green. **c**. *A. salmonicida* Hooke was stained with 300 nM 4′,6-diamidino-2-phenylindole (DAPI). **d**. Merge of b–c. (For interpretation of the references to colour in this figure legend, the reader is referred to the Web version of this article.)

After co-incubation of bacteria with cells that had been induced to release ETs, fewer bacterial colonies of *A. salmonicida* Hooke were recovered at 3 h after inoculation (1.27 × 10^6^ CFU/ml), though this difference was not significant when accounting for multiple comparisons (Fig. 6). However, when the ETs had been digested away by the addition of recombinant DNase I, the abundance of bacterial colonies remained similar to the inoculum (2.30 × 10^6^ CFU/ml at 3 h compared with 2.52 × 10^6^ CFU/ml at inoculation).

### 3.5 ETs released by other cells of myeloid origin

**Fig. 7:**
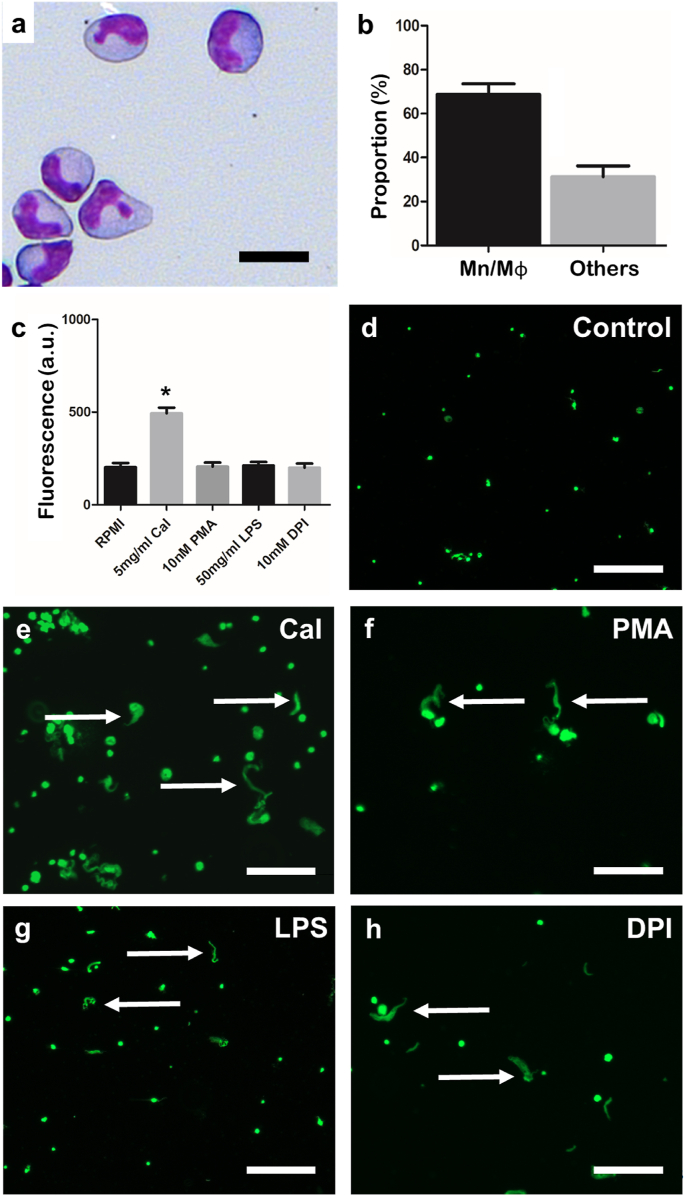
**Effects of previously characterised stimulants on extracellular traps (ETs) released from macrophage-enriched cell suspensions from Atlantic salmon.a.** Light microscopy image of cytospin slide of isolated mononuclear cells stained with Rapi-Diff II; scale bar, 20 μm. **b.** Bar chart showing the percentage of monocyte/macrophages (Mn/Mɸ) in the isolated cell population (mean ± SEM, n = 9). **c.** Bar chart showing fluorescence (mean ± SEM) of macrophage-enriched cell suspensions after incubation (1 h, 15 °C) with various proposed inducers and inhibitors of ETosis and stained with 5 μM Sytox Green showing cultures exposed to calcium ionophore (CaI) had significantly greater fluorescence compared to the untreated control (indicated by ∗; CaI: *t* = −7.0314, p = 0.0001, n = 5), which was not the case for the other compounds (phorbol 12-myristate 13-acetate [PMA]: *t* = −0.1033, p = 0.9202, n = 5; lipopolysaccharide [LPS]: *t* = −0.1841, p = 0.8585, n = 5; diphenyleneiodonium chloride [DPI]: *t* = 0.0819, p = 0.9367, n = 5). **d**–**h.** Fluorescence microscopy images of macrophage-enriched cell suspensions incubated with different compounds (1 h, 15 °C); scale bars, 100 μm. **d.** Untreated controls spontaneously released a low abundance of ETs. **e.** Incubation with 5 μg/ml CaI. **f.** Incubation with 10 nM PMA. **g.** Incubation with 50 μg/ml LPS. **h.** Incubation with 10 μM DPI. (For interpretation of the references to colour in this figure legend, the reader is referred to the Web version of this article.)

Cells in suspensions enriched for macrophages (68.7 ± 4.9%) were round and had a characteristic bean-shaped nucleus surrounded by a large volume of cytoplasm. After 1 h stimulation with 5 μg/ml CaI, fluorescence from the wells increased significantly (493.7 ± 76.7 a.u.) compared to untreated controls (202.5 ± 22.83 a.u.) (Fig. 7). Microscopy confirmed that the macrophages appeared to have released structures resembling ETs that stained positively with Sytox Green, which occasionally led to the formation of large clumps (Fig. 7). Macrophage-enriched cell suspensions exposed to 10 nM PMA, 10 μg/ml LPS, or 10 μM DPI showed no significant change in fluorescence, which is consistent with no increase in ET release and microscopy observations confirmed this (Fig. 7).

Regarding PBLs, the isolated cells in the enriched suspension were small and round with a large round nucleus, common characteristics that make it difficult to differentiate lymphoid and myeloid subsets. However, by using specific antibodies against surface markers of trout thrombocytes and MACS, salmon thrombocytes were successfully isolated (**Supplementary figure**). Exposure of these cells to 10 nM PMA or 5 μg/ml CaI resulted in non-significant increases in fluorescence from the suspensions compared to the untreated controls (**Supplementary figure**). Visualisation by fluorescence microscopy showed mainly non-viable and lysed cells in untreated controls and cell suspensions treated with CaI (i.e., intact nuclei), with just a few ‘streaky’ structures observed in the cell suspensions incubated with PMA and further experiments are needed to confirm whether these structures are ETs (**Supplementary figure**).

## 4 Discussion

Innate immunity is the first line of the fish host defence against pathogens, with neutrophil-like cells in particular playing a critical role in this response. These phagocytes contribute to antimicrobial defences by eliminating microbes by phagocytosis, undergoing oxidative burst and, in many vertebrates, through the release of chromatin by the mechanism of ETosis. This present study is the most comprehensive so far on the phenomenon of ET release by immune cells of Atlantic salmon, an important farmed species.

ETs consist of decondensed chromatin (DNA and histones) in close association with cytoplasmic granules containing proteases and anti-microbial peptides [32,33], and these structural features are conserved in vertebrates and invertebrates [16,[34], [35], [36], [37]]. Structures consistent with ETs were detected in enriched suspensions of neutrophil-like cells prepared from healthy salmon pre-smolts. The extracellular DNA scaffold of the ET was visualised by staining with nucleic acid-specific dye, Sytox Green, while these structures could be digested completely within minutes after exposure to the nuclease, DNase I. Immunofluorescently-labelled antibodies specific for histone H2A and histone H1 in association with DNA confirmed the extracellular DNA to be chromatin and thus of nuclear origin. Finally, IFAT also confirmed the presence of neutrophil elastase within the ET structure, which is a key diagnostic marker, and confirmed the extrusion of nuclear material together with cytoplasmic granules into the extracellular environment [3,32,33,[38], [39], [40], [41], [42], [43]].

The ETs released in highly enriched cell suspensions of Atlantic salmon neutrophil-like cells formed spontaneously, which is consistent with observations on similar cell suspensions isolated from rainbow trout [10] and mammals [33,[44], [45], [46], [47]]. Spontaneous release of ETs in cultures *in vitro* may be due to the unfavourable nature of these conditions, and fish immune cells are short-lived outside the host. Still, it was possible to stimulate ET release from the Atlantic salmon neutrophils by exposure to CaI, which induces the release of ROS, and this conforms with findings from other studies on immune cells from fish [10,18,19]. In mammals, CaI stimulates ET release via an NADPHox-independent cascade, by activating potassium channels and inducing mitochondrial ROS production [48,49]. Meanwhile, under the experimental conditions of this present study, PMA (a stimulant of ET release in many species, particularly mammals) did not cause a significant increase in ET release from the Atlantic salmon neutrophils and it seems to be only a weak stimulant in fish [10,18,19,22]. Moreover, although purified LPS caused a significant increase in ET release from Atlantic salmon neutrophils, this was to a far lesser extent than CaI. In contrast, purified LPS failed to induce ET release in carp phagocytes [24] and rainbow trout PMNs [10], whilst ET release was stimulated in tongue sole neutrophils [11] and carp phagocytes when exposed to a less pure preparation of LPS [24]. Lipopolysaccharides are sensed through toll-like receptor 4 (TLR-4) that activates the MyD88-dependent pathway and the transcription factor NF-κB involved in ROS formation, which underlies the stimulatory role of LPS in ET release in mammals [50]. However, TLR-4 in teleosts has been described only in catfish, zebrafish and other cyprinid species [51], thus perhaps explaining the at best modest effect of LPS on the induction of ET release from salmonid neutrophils. The stimulatory effects of LPS on ET release may be due to impurities found in this reagent [24]. DPI is used commonly to inhibit ET release because it inhibits the required ROS generated by NADPH oxidase. However, Van et al. [10] found DPI to be an inducer of ET release from PMNs isolated from rainbow trout. This present study found that DPI did not stimulate ET release from Atlantic salmon neutrophils, thus the initial findings of Van et al. [10] were not corroborated, though this may be in part due to differences in exposure concentrations and incubation times. Of note, Alarcon et al. [23] found increasing ET release was not associated with a significant increase in ROS in leukocyte preparations from salmon, while Yirong et al. [52] and Pijanowski et al. [53] have reported ROS generation to be important in ET release by carp neutrophils. Seemingly contrasting findings like these further underscores our lack of understanding of the underlying pathways of ET release in fish and highlights the need for further study to elucidate the mechanisms underlying ET release.

Exposure of Atlantic salmon neutrophils to live *A. salmonicida*, the Gram-negative bacterium responsible for furunculosis, led to increased ET release. This is consistent with observations on other fish hosts in response to bacteria, including tongue sole PMNs responding to *Edwardsiella tarda*, *Vibrio harveyi* and *Pseudomonas fluorescens* [16], turbot neutrophils responding to *P. fluorescens* [22]*,* and Atlantic salmon PMNs responding to the intracellular bacterium *Piscirickettsia salmonis* [23]. Furthermore, recombinant flagellin from *Yersinia ruckeri* was a potent inducer of ET release from rainbow trout PMNs [10]. As such, bacterial pathogens and antigens appear to be strong candidates as natural triggers of ET release from fish neutrophils *in vivo*, similar to the case for mammals [9,54,55] and invertebrates [36,37,56].

Interestingly, cells derived from larger fish (i.e., older) were less likely to release ETs spontaneously, as fluorescence values from spontaneous ET release in PMN suspensions associated inversely with fish mass. This observation may underlie at least some of the variation seen between individual fish (e.g., Van et al. [10]), and several studies of non-fish species have associated ageing with reduced capacity to form ETs under certain circumstances. In adult humans with chronic periodontal infection, neutrophils from older adults are less likely to form ETs [57], whilst Tseng et al. [58] reported ageing-associated impairment of ET release in mice both *in vitro* and *in vivo*, with the latter associated with marked bacteraemia in a model of *Staphylococcus aureus* infection [58]. In addition, differences in neutrophil yields and the likelihood to release ETs spontaneously in the Atlantic salmon could be linked to the considerable physiological changes associated with onset of smoltification in these animals, which may occur at ca. 120 g and significantly impacts immunity [59]. Further experiments with closely genetically-related fish cohorts maintained under identical culture conditions would be warranted to confirm age-related effects on ET release in fish.

One focus of the present study was to assess the interaction between bacteria and ETs released by Atlantic salmon neutrophils. ETs act as part of the innate response to counter bacterial invaders by trapping bacteria to prevent dissemination around the host and/or exerting direct antimicrobial action through bringing trapped microbes into contact with the decorating proteins that can exert such activities [32]. The close interaction between ETs and bacteria was apparent by fluorescence microscopy. A trapping assay was performed to test whether the ETs from Atlantic salmon neutrophils trapped or inactivated *A. salmonicida*, where the bacteria were brought into close contact with intact ETs or ETs that had been digested away with DNase and then the abundance of CFU determined after 3 h incubation. Fewer CFU were recovered from cultures containing intact ETs, which may indicate trapping of the bacteria within the mesh of the ET or inactivation of bacteria by ET components. The antimicrobial components of digested ETs would still have been present in the control cultures but these may have been unable to act against the bacteria due to being distributed throughout the medium and not brought into close proximity with the bacteria for a sufficient time. Though the reduction in CFU due to the ETs just failed to reach statistical significance, this observation warrants further investigation and other studies have found ETs released by fish to exert trapping and antimicrobial actions. Entrapment of bacteria and inhibition of replication has been reported for turbot neutrophils incubated with *P. fluorescens* [22], and carp neutrophils incubated with *Aeromonas hydrophila* [60].

Finally, the capabilities of other phagocytes to release ETs was examined in Atlantic salmon, specifically for monocyte/macrophages and thrombocytes, as mammalian eosinophils, basophils and macrophages have shown ET release previously [[61], [62], [63], [64], [65], [66], [67], [68]], whilst carp macrophage preparations have also been reported to contain ETs [24]. These other cell types share a common myeloid progenitor with neutrophils and so the ability to release ETs may be retained [69]. Consistent with Pijanowski et al. [24], the salmon macrophage suspensions contained ET-like structures that stained positively with Sytox Green after, in our case, stimulation with CaI. Though unlikely, given the purity of the preparations, it cannot be excluded that the ET-like structures observed could have derived from other cell types contaminating the suspension. Thrombocytes were also examined for their ability to release ETs, as these cells are nucleated and phagocytic in fish [70], unlike their mammalian counterparts the platelets. Thrombocytes are the most abundant blood cell type after the erythrocytes in most teleosts [71] and these cells contribute to the immune response of lower vertebrates by performing phagocytosis and expressing immune-relevant genes and enzymatic activities [31,72]. Indeed, thrombocytes from cyprinid species and flounder play roles to counter bacteria [73,74], whilst in mice platelets interact with neutrophils to cause ETs to be released *in vivo* into the vasculature [55]. Salmon thrombocytes, isolated by MACS expressed conserved membrane glycoprotein domains similar to those on rainbow trout thrombocytes [31], were incubated with PMA and CaI; however, there was little evidence for ET release besides some ‘streaky’ structures in fluorescence microscope images from PMA-exposed cells.

Fluorescence assays were used to quantify ET release from cells throughout this present study, but these assays may be influenced by dead or membrane-compromised cells in the suspensions that may be dying by apoptosis or necrosis, as nuclei from these cells will also stain positively with Sytox Green. Controlling for cells that die by these other pathways is difficult and serves to emphasise the importance of including microscopy observations to support findings relying on fluorescence data quantification of ETs. A further limitation of the present study is that all experiments were performed *in vitro*, and *in vivo* trials will be essential if the roles and functions of ETs in the fish innate response are to be fully understood.

## 5 Conclusion

To conclude, this present study demonstrates that ETs are released by salmon phagocytes, including neutrophils, thus providing a foundation for further study. The ET structures and the modulators of their release are consistent with reports for immune cells isolated from other species of fish, thereby supporting the evolutionarily conserved nature of this defence response. A deeper understanding for the roles and functions of ETs within innate immunity of fish hosts, and their interaction with microbial pathogens, may open new avenues towards protecting cultured stocks against infectious diseases.

## Funding

This study was funded by a grant awarded equally by 10.13039/501100000268BBSRC and NERC under the Sustainable Aquaculture Initiative (grant reference: BBM026132/1).

## CRediT authorship contribution statement

**Neila Álvarez de Haro:** Data curation, Formal analysis, Investigation, Methodology, Writing – original draft, Writing – review & editing. **Andre P. Van:** Methodology, Writing – review & editing. **Calum T. Robb:** Methodology, Resources, Writing – review & editing. **Adriano G. Rossi:** Conceptualization, Funding acquisition, Methodology, Project administration, Resources, Writing – review & editing. **Andrew P. Desbois:** Conceptualization, Data curation, Formal analysis, Funding acquisition, Methodology, Project administration, Resources, Writing – original draft, Writing – review & editing.

## Declaration of competing interest

The authors confirm they have no known conflicts of interest.
