# Supplementary material for: Release of chromatin extracellular traps by phagocytes of Atlantic salmon, Salmo salar (Linnaeus, 1758)
Source: Fish Shellfish Immunol. 2021 Dec;119:209–19. doi: 10.1016/j.fsi.2021.08.023 (PMC8653909; doi:10.1016/j.fsi.2021.08.023)
Supplement: Multimedia component 1 [file mmc1.docx]

**Supplementary figure**


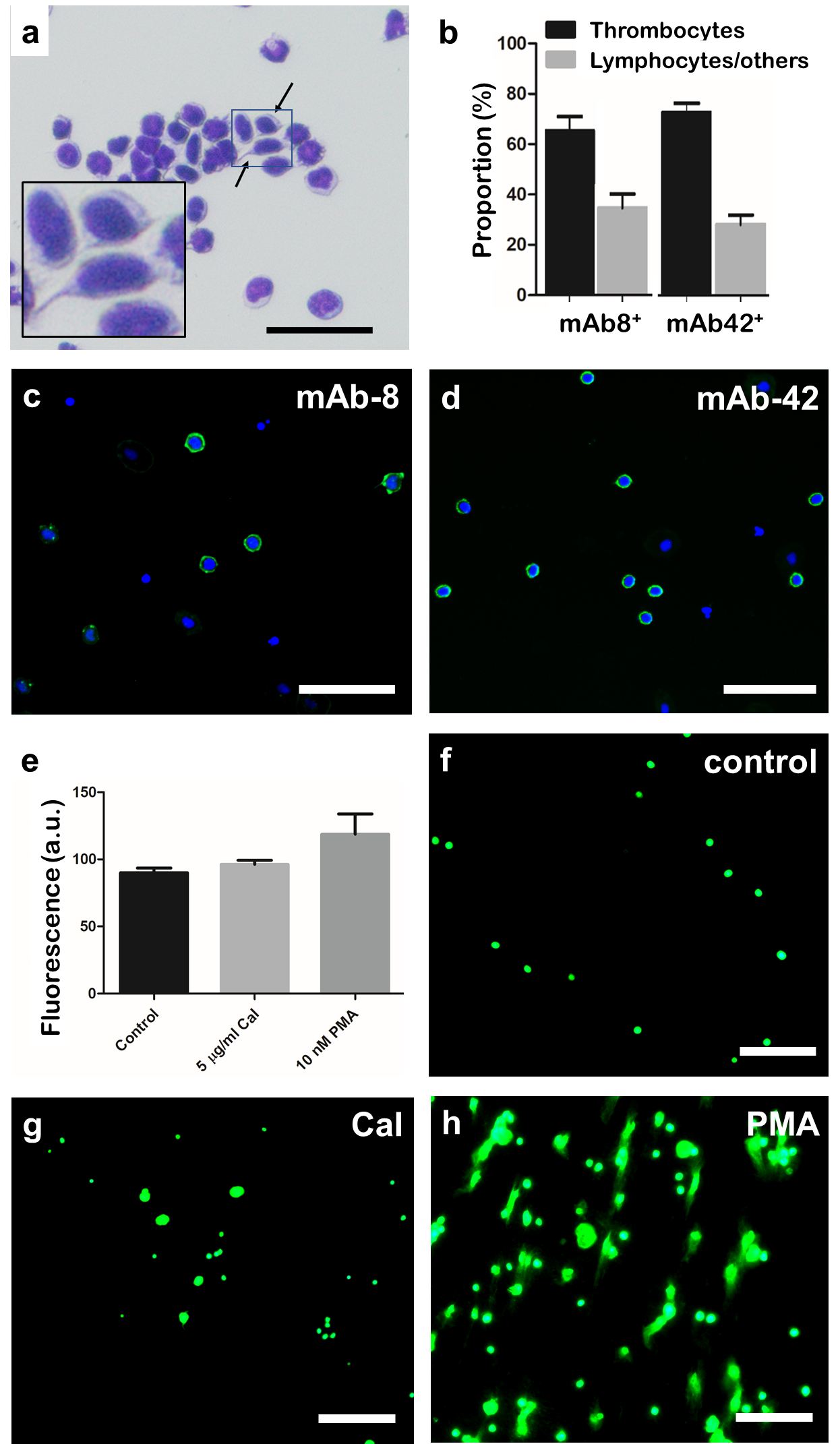


**Supplementary figure. Effects of previously characterised stimulants on extracellular traps (ETs) released from thrombocyte-enriched cell suspensions** **from Atlantic salmon.** Peripheral blood leukocytes were sorted to positively select for thrombocytes and incubated with proposed inducers of ETosis (1 h, 15ºC). **a.** Cytological spin slide of the isolated cells collected as described in Section 2.1 and stained with Rapi-Diff II. The cells presented a basophilic mononuclear morphology, with a round central nucleus or the characteristic spindle shape nucleus (arrows); scale bar, 50 µm. **b.** Before immunomagnetic sorting, 65% of cells in the suspension stained positively with mAb-8, whilst 72% stained positively with mAb-42. **c.** Microscopic image of thrombocytes stained with mAb-8 (Köllner et al., 2004) and conjugated Alexa-488, with nuclei counterstained with 4′,6-diamidino-2-phenylindole (DAPI). **d.** Microscopic image of thrombocyte membrane glycoproteins stained with mAb-42 and conjugated Alexa-488, with DAPI-counterstained nuclei. **c−d** Scale bars, 50 µm. **e.** Bar chart showing fluorescence (mean ± SEM; n = 3) of salmon thrombocyte cell suspensions (enriched by magnetic-activated cell sorting) incubated with 5 µg/ml calcium ionophore (CaI) or 10 nM phorbol 12-myristate 13-acetate (PMA). There were no significant differences between the CaI or PMA treatments and the untreated controls (CaI: *t* = -1.3571, p = 0. 2463, n = 3; PMA: *t* = -1.8360, p = 0. 1402, n = 3). **f−h.** Fluorescence microscopy images of thrombocyte-enriched cell suspensions incubated with proposed inducers of ETosis and stained with 5 µM Sytox Green; scale bars, 100 µm. **f.** Untreated controls. **g.** Cells incubated with 5 µg/ml CaI. **h.** Cells incubated with 10 nM PMA.
